# Gleanings from Our Exchanges

**Published:** 1872-05-15

**Authors:** 


### Gleanings from Our Exchanges.

### THE RELATIONS BETWEEN HÆMOPTYSIS AND PULMONARY TUBERCULOSIS.

A CLINICAL LECTURE BY PROF. SKODA.

(*Translated from the Annales et Bulletin de la Societe de Med. de Gand, for the Boston Med. and Surg. Jour.*)

Prof. Niemeyer has recently assigned to hæmoptysis an importance entirely unlike that which it formerly was held to possess. He believes that tuberculosis is caused by the hæmoptysis itself, maintaining that the blood arrested in the bronchial tubes and in the air-cells after a hæmorrhage, gives rise to a chronic inflammation, and that on this depend the febrile state and the other symptoms of phthisis. If the blood thus retained in the minute bronchi and in the air-cells really possessed such an influence, and could excite such an inflammatory state, we ought to expect that the same result would follow hæmorrhages which attend cardiac disease. Now, no such condition occurs in the course of that affection. Where an hæmoptysis takes place in patients whom we consider to be tuberculous, and who die during the hæmorrhage or soon after, we do not generally find any arrest of accumulated blood in the bronchi and air-cells; while if death occurs after a hæmorrhage in diseased heart, there is found a collection of blood in the lung. The hæmorrhagic infarctus very rarely presents itself after the hæmoptysis of tuberculosis, and is an exceptional occurrence in cardiac disease. But it is this very thing which would determine the conditions of a chronic inflammation! I have never seen such a result. Doubtless, if accumulated blood does remain, a moderate reaction occurs, in the course of which only the normal changes of the blood take place; that is, it coagulates, becomes encysted, and forms the infarctus alluded to, but never progresses to suppuration. Such a hæmorrhagic infarctus may last months and years, growing smaller and smaller, and finally disappearing altogether. The blood-globules undergo a metamorphosis by which the black pigment is the result, or else disappear by fatty degeneration. The fluid elements, which become separated from the rest, are reabsorbed; the dark-coloring matter is left, and if the hæmorrhagic infarctus continues any length of time, it remains as black patches in the substance of the lungs. According to this view, then, the observations relative to the effusion of blood in the lungs in the course of disease of the heart, accord so little with the theory of Prof. Niemeyer, that one is forced to confess that this hypothesis is untenable.

According to the investigations which have been made in the living subject and upon the cadaver, it is very probable that the hæmoptysis which occurs in pulmonary tuberculosis before and during its development, has its seat in the mucous membrane of the bronchi, and not in the air-cells. If the blood came from the latter, it would certainly be very difficult to explain the rare occurrence of the hæmorrhagic infarctus; but since it comes from the bronchial mucous lining, it is easy to see that none remains as a plug, but that it is expelled by coughing. I can state positively that in cases in which death occurs in the course of an hæmoptysis, it is the rare exception to find blood in the bronchial tubes, but that it is found rather in the larynx and the trachea; because, by the cough and the contraction of the bronchi, it is at once drawn forward and expelled.

So, too, I cannot accept the theory that the hæmoptysis may give rise to serious after effects. Such a result can be only in cases in which the hæmorrhage occurs in a lung tissue already diseased, especially in cavities from which the blood cannot be evacuated; and it is possible that the morbific properties peculiar to the cavities themselves, contribute thus to develop a more active irritation. It is, moreover, to be noted that the blood is not specially irritant to the tissues; for example, a hæmorrhage into the subcutaneous tissues after a blow does not produce any marked irritation, as we very well know, but it is generally quickly reabsorbed; so there is no reason for supposing that the blood is so irritant in a tuberculous patient as to favor the development of the symptoms of the disease. Nevertheless, I attribute a great importance to hæmoptysis, but only as a symptom indicating that the disease is present, or that it is in process of development.

Another question here presents itself. When directly consequent upon an acute pneumonia, there remains some of the inflammatory product in the lungs, a chronic pneumonia is said to exist. This deposit differs materially from those peculiar to the disease which we call tuberculosis. The former can remain months and years without lighting up mischief, while in tuberculous disease cavities become formed with the greatest ease. I see, therefore, an important distinction between the two diseases, and it is useless to apply terms in common which may give rise to confusion.

Therefore we see that hæmoptysis is not the cause of consecutive disease of the lung; on the contrary, the cause of the pulmonary disease resides elsewhere, and the hæmorrhage is only a symptom of a morbid predisposition which subsequently manifests itself under the form of tuberculosis.

Hæmoptysis likewise proceeds without doubt from other causes—cardiac disease, for example. Moreover, certain cases of hæmoptysis occur independent of disease of the heart, having no connection, indeed, with eventual pulmonary disease, cases in which the hæmorrhage frequently recurs, but with no serious pulmonary affection consequent. But such instances are rare, and are sometimes dependent upon a tuberculous degeneration limited to a single point in the lung, which, once diseased, never returns perfectly to its normal state, and becomes the seat of hæmorrhages which recur from time to time. Other cases, also, are observed in which the extravasation of blood proceeds solely from the capillaries or from dilated veins, among which aneurisms by anastomosis are found. Doubtless a metamorphosis of the pulmonary parenchyma can thus give rise to a serious attack of hæmoptysis; these attacks may recur, and yet no tuberculosis ever result; when the hæmorrhage ceases, the patient regains his previous health; debility may result, as in other cases of hæmorrhage, but farther than this there exists no other symptom worth noting.—*The Doctor, London.*

Dr. Voisin on the Treatment of Epilepsy by Copper and Zinc.—In the *Wien. Med. Zeit.* No. 9, Dr. Voisin says that the definitive cure of epilepsy is at present so doubted by a great crowd of medical observers, that he holds it useful to quote from Herpin, of Geneva, who left behind him notes on this subject, a series of cases when the disease was cured for a shorter or longer time. He thinks it the more important to do so at present because just now bromide of potassium is so prodigiously praised in the treatment of epilepsy, that one is inclined to consider the former remedies as having all been useless and without power, whilst clinical experience shows us every day that bromide of potassium is very far from curing all epilepsies,—and especially the essential ones—or even to do them much good; and that, indeed, several patients are by its means excited extremely; whilst in others its supposed power of lowering sexual emotions is null; indeed, in some cases the sexual irritation is increased. Dr. Herpin, of Geneva, was not acquainted with the anti-epileptic properties of bromide of potassium. He treated his patients chiefly with preparations of lactate of zinc, ammonio-sulphate of copper, hyoscyamus, and artimisia. In a young girl who, when aged thirteen, had contracted essential epilepsy, Herpin treated her ten years later, after that she had suffered 111 times from *grand mal*, and on numerous occasions from fainting-fits and vertigo. After the patient had been treated with lactate of zinc, hyoscyamus, and artimisia, she was cured when the last remedy was raised to 10 grammes. The experiment was continued a year longer, and since then the girl has remained quite free from the disease, and healthy. The second patient was twenty-one months old when brought to Herpin; and the disease was hereditary in this case, and fully developed. Attacks came on 25 times a month, and 150 had already been counted. Treatment consisted in the use of lactate of zinc, ammonio-sulphate of copper, hyoscyamus, and artimisia. The disease got milder by the use of the copper, and disappeared entirely when artimisia was used; and since 1858 the child, who was now a man, was quite healthy. The third patient came to Herpin at the age of eleven years, after having for six months had 38 epileptic attacks, and numerous attacks of vertigo. Four years were required of treatment by the above-mentioned remedies; and here it was the copper which conquered the disease; Herpin at the same time made the lad practice gymnastic exercise, and take long walks in the open air. The fourth observation relates to an epileptic who was brought to Herpin when eighteen years old. He had only two severe fits. He was treated by copper, and the disease was quenched. The fifth case was in a girl 19 years of age, who had been epileptic a year. She had had five attacks, each lasting two hours. Treatment with lactate of zinc, undertaken for a year cured the girl; and since then she has not had a trace of the disease. The sixth case was that of a girl eleven years old; and she had suffered since her seventh year from epilepsy and vertigo; 53 times from the former, *grand mal*. She was treated with the lactate of zinc for a year, and cured definitely. The seventh case was that of a boy eight years old, whose uncle by the mother's side was insane; had his first attack in July, 1850, when four years of age. He was first put under the water-cure, and then on belladonna. Under these his complaint became milder; and lactate of zinc cured the vertigo. He has been quite well since 1854.—*The Doctor*.

New Attempts at Inoculation of Grey Tubercle.—Dr. Serafuro Biffi, and Dr. A. Verga (*Gazz. M. C. Lomb.*, August, 1871), say that the argument of the inoculability of tuberculosis is far from having exhausted the attention and patience of cultivators of experimental pathology. Everywhere is repeated the experiment of inoculating animals of different species, and in different parts of their organism, not only with grey tubercle, but with the so-called cheesy matter, and other morbid products; lastly with inert substances. But up to this time the question has not been settled so as to satisfy the students, so that they are divided into two camps, in which the most opposite opinions are held. Experiments were made by these gentlemen on two mules, one cow, two pigs, and one dog, in the veterinary school of St. Francisco, near Milan. In all these seven animals they had inoculated under the skin human grey tubercle, which was triturated and reduced to a soft pultaceous matter by the addition of distilled water. This was introduced amidst the subcutaneous cellular tissue, through a little puncture in the cutis, which was then reunited with a point of suture. In another case the matter, much diluted with distilled water, and passed through a piece of gauze, was injected by means of one of Pravaz's syringes. In one way or other the injection was always practiced on each animal at least in two distinct points, in the neck and the thigh. It was noticed that some days after the operation, where it had been practiced, the connective tissue became inflamed, and there was a swelling noticed, which afterward assumed the appearance of a nodule, then opened, and gradually the wound cicatrized. The pigs, the dogs, and the mules, were killed about three months after inoculation, and the cow after two months. All these animals, after the operation, had been kept in good conditions of health, and appeared to enjoy good health. The accurate autopsies showed that there was no tubercle either in the lungs or any other parts of these animals.—*The Doctor*.

Aneurism of Popliteal Artery by Flexion of the Knee.—Dr. Larondelle (*Presse Med. Belge*, Oct. 8) says that in 1838 Malgaigne indicated flexion of the leg on the thigh as a method of curing popliteal aneurism, having observed that when the forearm was much flexed on the arm, the pulsations of the radial artery ceased. Thierry, in 1852 (*Gaz. d' Hop.*), had indicated that forced flexion would cure diffuse aneurism of the branchial artery. Ernest Hart, Shaw, and Spence, in England, have cured aneurism of the popliteal artery by flexion. Anatomically experimenting after death when the leg is at right angles with the thigh, the popliteal artery becomes flexuous in the portion seated beneath the line of articulation. The flexuousities it describes are very small, and close together. At the same time the artery, at the level of the line of articulation, becomes flattened in the lateral direction, and forms an obtuse angle, of which the vertex is directed inwards. As we augment the flexion of the leg, the angle of flexion of the vessels becomes more acute, its flattening more and more marked, and the flexuousities approach each other more nearly. Above the line of flexion the artery preserves its normal direction. To ascertain the influence which the flexion exercises upon a current of liquid in the popliteal artery, recourse was had to injection of water into the femoral artery, after first cutting the tibial artery behind the internal malleolus. The jet which issued forth from the posterior tibial when the limb was straight, became arrested entirely when the leg was greatly flexed on the thigh. When flexion can be carried far enough, it is then an excellent method of treatment.—*The Doctor*.

A Chair for Hygiene Established in the University of Vienna, Austria.—The Sanitary Counselor of Austria, has declared it a necessity the founding of a chair for hygiene. It will be taught as a branch of physiology, and lectures will be given bearing on this as well as pathology and therapeutics. The subject must not be studied alone by sanitary officials, but each physician must interest himself in it in order to keep pace with the progress, not only of the medical science, but the natural sciences. The interests can be best advanced when physicians unite their knowledge to that of those interested in these subjects, as, for instance, physiologists, chemists, engineers, manufacturers, refiners, etc. They all have a common interest to advance the rule of hygiene, and to regulate evils that do not come directly under the observation of the physician. There must be societies, unions, periodic meetings—a central point from which to radiate and to which to bring the varied experience of each; this must be published, the people must become interested in the subjects discussed, and a paper dedicated to them, as to political and other subjects. This has been successfully tried in London; the people are zealous in furthering the cause established and directed by the medical fraternity.

